# Endothelial Injury as a Potential Contributor to Cardiovascular Toxicity in Hematologic Malignancies

**DOI:** 10.3390/diseases14090342

**Published:** 2026-09-16

**Authors:** Aladin Altić, Nikola Jovanovic, Dario Vucic, Nikolaos Katsivelos, Lydia Giannakou, Jongyeob Kim, Ali Maaz, Una Tonkovic, Aleksandar Sič, Marko Baralic

**Affiliations:** 1 Department of Internal Medicine, Albert Einstein College of Medicine, Jacobi Medical Center/North Central Bronx Hospital, New York, NY 10467, USA; nikola92.jovanovic92@gmail.com (N.J.); katsiven@nychhc.org (N.K.); giannakl@nychhc.org (L.G.); crossing96@gmail.com (J.K.); alimaazkhosa@gmail.com (A.M.); 2 Regional Health Center Siroki Brijeg, 88220 Siroki Brijeg, Bosnia and Herzegovina; 3 Faculty of Medicine, University of Belgrade, 11000 Belgrade, Serbia; unaunattt@gmail.com (U.T.); baralicmarko@yahoo.com (M.B.); 4 Nephrology Clinic, University Clinical Center of Serbia, 11000 Belgrade, Serbia

**Keywords:** oxidative stress, vascular dysfunction, cardio-oncology, biomarkers

## Abstract

Cardiovascular toxicity is an important complication of treatment for hematologic malignancies, but it is often discussed mainly in terms of direct myocardial injury. Endothelial injury may provide a common link between several cardiovascular complications seen in these patients. Endothelial dysfunction may already be present because of the malignancy itself and can be further increased by anticancer treatment, immune activation and haematopoietic stem cell transplantation. Oxidative stress, reduced nitric oxide availability, inflammation, increased vascular permeability and activation of coagulation can shift the endothelium from a protective to a pro-inflammatory and prothrombotic state. These changes could contribute to hypertension, thrombosis, atherosclerotic disease, coronary microvascular dysfunction, arterial stiffness and myocardial dysfunction. The role of endothelial injury is particularly relevant with BCR-ABL inhibitors, proteasome inhibitors, CAR-T-cell therapy, bispecific antibodies and transplantation, although the mechanisms and strength of evidence differ between treatments. Several circulating biomarkers and tools, including angiopoietin-2, von Willebrand factor, soluble thrombomodulin, adhesion molecules and the Endothelial Activation and Stress Index, may help identify endothelial injury, but their clinical roles still remain unclear. This review examines endothelial injury across hematologic malignancies and their treatments and discusses its role in cardiovascular toxicity, current approaches to vascular protection and the main gaps that need to be addressed before endothelial assessment can become part of routine cardio-oncology care.

## 1. Introduction

Advances in the treatment of hematologic malignancies have substantially improved survival, but they have also increased the importance of treatment-related cardiovascular toxicity [1]. Current therapies are associated with a wide range of cardiovascular complications, including heart failure, hypertension, arrhythmias, arterial and venous thrombosis and ischemic vascular disease. These complications are seen not just with conventional chemotherapy but also with targeted agents, proteasome inhibitors, immunomodulatory therapies, cellular therapies and hematopoietic stem cell transplantation (HSCT) [2].

Cardiotoxicity has traditionally been viewed mainly as a result of direct cardiomyocyte injury, particularly after anthracycline exposure. However, the vascular endothelium is also an important target of anticancer therapy. Endothelial injury can impair nitric oxide signaling, increase oxidative stress and inflammation, alter vascular permeability and promote a prothrombotic state. These changes can affect both large and small vessels and may help explain the different cardiovascular complications seen during and after cancer treatment. This may be particularly important in hematologic malignancies, where endothelial injury can result from several factors at the same time [3,4]. The malignancy itself, systemic inflammation, anticancer treatment and immune activation can all affect endothelial function. The role of endothelial injury is especially clear after HSCT, where it contributes to complications like sinusoidal obstruction syndrome, transplant-associated thrombotic microangiopathy, capillary leak syndrome and graft-versus-host disease. Endothelial activation is also involved in cytokine release syndrome and other complications of CAR-T-cell therapy. Endothelial injury may be a common link between cardiovascular complications that are usually considered separately [5,6].

The aim of this review is to evaluate whether endothelial injury can provide a common mechanistic framework for understanding cardiovascular toxicity in hematologic malignancies. We focus on how disease- and treatment-related endothelial damage translates into cardiovascular complications, and examine its potential value for risk assessment, monitoring and prevention.

## 2. Endothelial Injury as a Common Pathophysiological Pathway

In this review, endothelial injury is used as an overarching term encompassing structural and functional endothelial alterations. Endothelial dysfunction, activation, increased permeability and senescence describe specific, frequently overlapping manifestations of this process.

Vascular endothelium is a dynamic interface that regulates vascular tone, permeability, inflammation, leukocyte trafficking, platelet activity, coagulation and fibrinolysis [7]. In physiological conditions, endothelial nitric oxide synthase (eNOS)-derived nitric oxide (NO) maintains vasodilation and exerts anti-inflammatory and antithrombotic effects. Endothelial injury disrupts this equilibrium and shifts the vascular surface toward a vasoconstrictive, inflammatory, permeable and prothrombotic phenotype [8]. Reduced NO bioavailability, increased oxidative stress and endothelial activation are therefore key features of endothelial dysfunction and provide an early mechanistic link between vascular injury and cardiovascular disease [9].

Oxidative stress and inflammation appear to form a mutually reinforcing axis of endothelial injury. Increased generation of reactive oxygen species (ROS) reduces NO bioavailability and impairs endothelial-dependent vasodilation, while inflammatory signaling promotes endothelial activation and the expression of adhesion molecules, including vascular cell adhesion molecule-1 (VCAM-1) and intercellular adhesion molecule-1 (ICAM-1) [10,11]. These changes facilitate leukocyte adhesion and transmigration and further amplify local vascular inflammation. At the same time, endothelial activation promotes platelet adhesion and coagulation, including release of von Willebrand factor (vWF) and produces a shift from the normally antithrombotic endothelial surface toward a procoagulant state [12,13].

In the setting of malignancy and anticancer therapy, these mechanisms rarely occur in isolation [4]. Direct endothelial cellular injury, ROS generation, inflammatory and innate immune activation, impaired NO signaling, increased vascular permeability and altered coagulation can converge to produce both macrovascular and microvascular dysfunction [4]. Persistent endothelial activation may lead to endothelial apoptosis or senescence and impaired vascular repair, potentially converting an initially reversible functional abnormality into sustained vascular injury [14]. This is especially relevant to hematologic malignancies, in which endothelial cells may be exposed simultaneously to the biological effects of the malignancy, systemic inflammation, cytotoxic or targeted therapy, immune activation and transplantation-related insults [15,16]. The concept is well established in hematopoietic cell transplantation, where otherwise distinct complications like sinusoidal obstruction syndrome, transplant-associated thrombotic microangiopathy, capillary leak syndrome and graft-versus-host disease share endothelial activation and injury as an important component of their pathophysiology [6,17].

**Figure 1:**
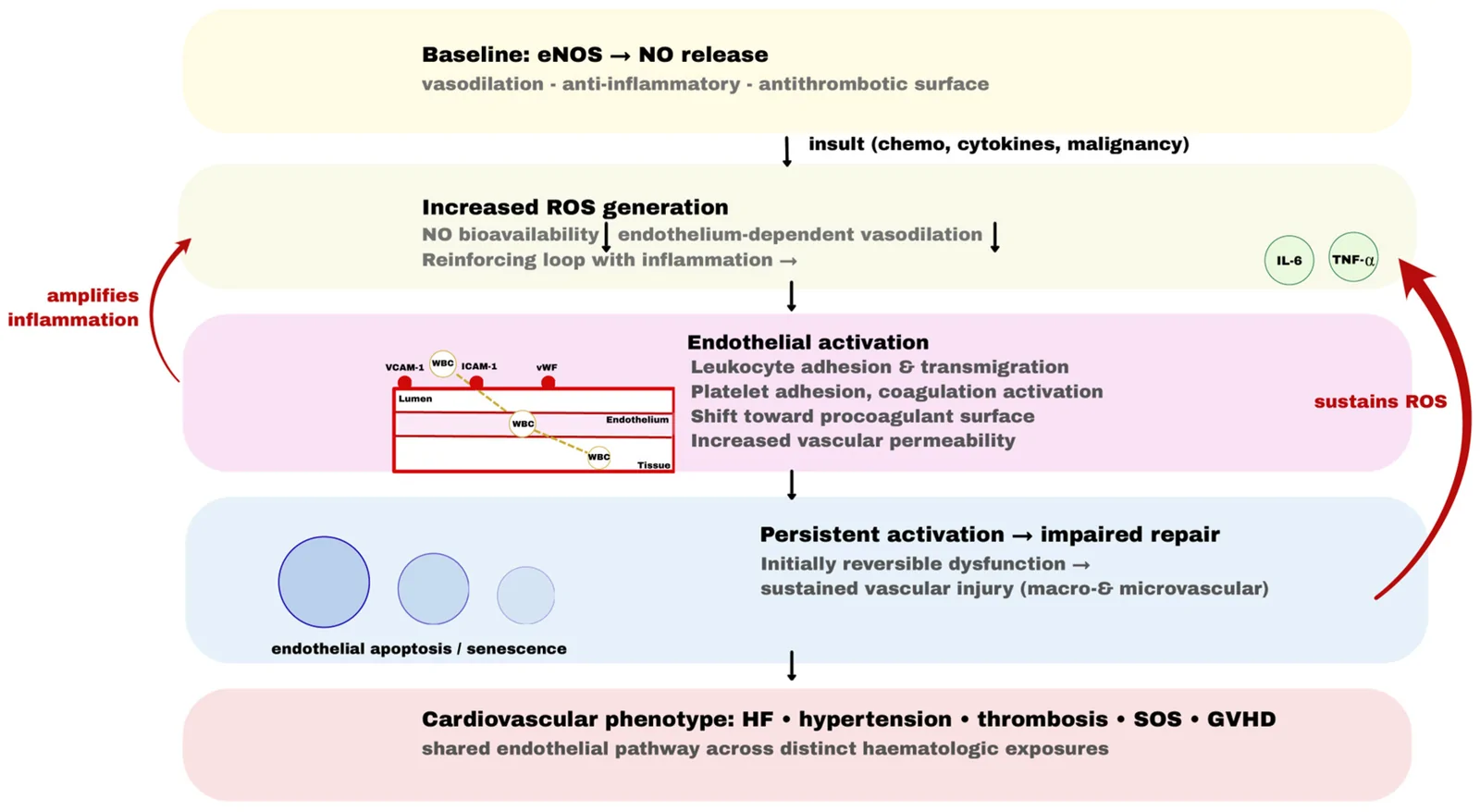
Main pathways involved in endothelial injury and cardiovascular toxicity. Oxidative stress, reduced nitric oxide bioavailability, inflammation, endothelial activation, increased permeability and procoagulant changes form interconnected pathways that may progress from potentially reversible dysfunction to persistent macrovascular and microvascular injury. Feedback loops between inflammation, oxidative stress and endothelial activation may amplify this process. eNOS, endothelial nitric oxide synthase; NO, nitric oxide; ROS, reactive oxygen species; HF, heart failure; SOS, sinusoidal obstruction syndrome; GVHD, graft-versus-host disease.

Therefore, endothelial injury should not be seen as a single downstream adverse effect, but as a joint biological response to heterogeneous insults. Oxidative stress, loss of NO signaling, inflammatory activation, endothelial permeability, leukocyte and platelet adhesion and procoagulant transformation form a pathway capable of translating different hematologic diseases and therapeutic exposures into clinically important cardiovascular phenotypes [4,18]. These interconnected pathways and their feedback relationships are summarized in Figure 1.

## 3. Disease-Related Endothelial Injury in Hematologic Malignancies

Endothelial dysfunction in patients with hematologic malignancies cannot be attributed only to anticancer treatment. The disease itself can change vascular function through inflammation, activation of coagulation, abnormal interactions between blood cells and the vascular wall and release of cytokines and extracellular vesicles [19,20]. The importance of these mechanisms differs between malignancies, but they suggest that some patients may already have endothelial dysfunction before treatment begins.

The strongest evidence comes from myeloproliferative neoplasms (MPNs), where thrombosis is a major cause of morbidity and mortality. Endothelial cells in MPNs can develop a pro-inflammatory and prothrombotic phenotype, with changes in von Willebrand factor (vWF), P-selectin, E-selectin, soluble thrombomodulin and other proteins involved in adhesion and coagulation. JAK2V617F is especially important in this process. Endothelial expression of JAK2V617F can increase P-selectin exposure and strengthen leukocyte-endothelial interactions, creating a vascular surface that favors thrombosis. JAK2V617F-positive endothelial cells have also been identified in some patients with MPNs, particularly in the splanchnic circulation [21,22,23].

The endothelium does not necessarily need to carry the mutation itself to be affected. JAK2V617F-positive cell-free DNA and exosomes released by malignant cells can act on endothelial cells and activate inflammatory and coagulation pathways. These extracellular materials can increase expression of adhesion molecules and coagulation-related genes and activate pathways such as cGAS-STING and TLR9. More recent clinical and experimental evidence also shows increased endothelial inflammation, permeability, vWF production and abnormal cellular adhesion in JAK2V617F-positive MPNs, particularly in patients with splanchnic vein thrombosis [21,24].

A different form of endothelial injury can occur in acute leukaemia. In patients with acute myeloid leukaemia and marked hyperleukocytosis, leukostasis is not simply a mechanical obstruction caused by large numbers of circulating blasts. Leukaemic cells interact directly with endothelial cells through selectins, integrins and other adhesion molecules. Inflammatory cytokines further activate the endothelium and increase these interactions. Adherent blasts can then migrate through the endothelial barrier, leading to vascular leakage, local tissue infiltration, impaired microvascular perfusion and haemorrhage. This is particularly important in the pulmonary and cerebral circulation, where leukostasis can cause severe organ dysfunction [25,26].

The relationship between malignant cells and the endothelium also extends beyond acute vascular complications. Endothelial cells form part of the bone marrow microenvironment and communicate continuously with hematologic tumour cells. Malignant cells can release extracellular vesicles containing proteins, lipids, DNA, RNA and microRNAs that alter the behaviour of surrounding cells [19]. In acute myeloid leukaemia, for example, extracellular vesicle-mediated transfer of VEGF-related signals can change endothelial metabolism and promote vascular remodelling. These changes may support malignant-cell survival and treatment resistance, showing that the interaction between the tumour and the endothelium can work in both directions [27].

Multiple myeloma provides another example of this close interaction. The bone marrow in myeloma is highly vascular, and endothelial cells are involved in the angiogenic environment that supports plasma-cell growth [28]. Myeloma cells, stromal cells, immune cells and endothelial cells exchange cytokines and extracellular vesicles within this environment [29]. At the same time, patients with multiple myeloma may already have a pro-inflammatory and procoagulant state before treatment, making it difficult to separate disease-related endothelial dysfunction from the effects of immunomodulatory drugs, proteasome inhibitors, transplantation and newer immune therapies [30]. Endothelial injury is therefore increasingly recognized as part of the wider biology of multiple myeloma, although its direct contribution to cardiovascular disease remains less clearly defined than in MPNs.

Evidence in lymphoid malignancies is less direct. Systemic inflammation, abnormal cytokine signalling, coagulation activation and interactions between malignant cells and the tumour microenvironment can affect endothelial cells [31], but most clinical data on vascular dysfunction in lymphoma come from patients who have also received potentially vascular-toxic treatments. It is therefore difficult to determine how much endothelial injury is caused by the lymphoma itself. This distinction is important when considering cardiovascular toxicity because disease-related and treatment-related effects often occur together rather than separately. Extracellular vesicles may provide one link across these different diseases. They allow malignant cells to influence endothelial cells without direct cell-to-cell contact and can carry inflammatory, angiogenic and procoagulant signals through the circulation. Their role has been described across leukaemia, lymphoma, multiple myeloma and MPNs, although much of the evidence remains experimental [19,32].

Endothelial injury may therefore already be present when anticancer treatment begins. In some diseases, such as MPNs, it is closely linked to thrombosis, while in acute leukaemia it can contribute to severe microvascular injury and leukostasis. In other malignancies, the contribution is less clear but may still add to the vascular effects of treatment. A multiple-hit model is therefore useful: disease-related endothelial dysfunction creates the background, while anticancer therapy, immune activation, transplantation and conventional cardiovascular risk factors add further vascular stress [4,33].

## 4. Treatment-Related Endothelial Injury in Hematologic Malignancies

Treatment of hematologic malignancies can affect the endothelium in different ways. Some drugs cause direct endothelial damage, while others interfere with pathways involved in vascular tone, endothelial survival, inflammation or coagulation [34]. The importance of endothelial injury also differs between treatments. In some cases, like BCR-ABL inhibitors and HSCT, there is strong evidence for direct vascular involvement, while for other therapies the endothelial contribution is less clearly defined [35].

Anthracyclines are best known for their direct effects on cardiomyocytes, but their toxicity is not limited to the myocardium. Doxorubicin can damage endothelial cells, reduce nitric oxide availability and promote endothelial apoptosis and senescence. This impairs vasodilation and may affect the coronary microcirculation. Endothelial dysfunction may therefore develop alongside direct myocardial injury and contribute to the wider cardiovascular effects of anthracycline treatment [36,37,38].

The vascular effects of BCR-ABL tyrosine kinase inhibitors are particularly important. Imatinib has a relatively favorable cardiovascular profile, while newer agents show clear differences in vascular toxicity. Nilotinib and ponatinib are associated with arterial occlusive events, including myocardial infarction, stroke and peripheral arterial disease. Dasatinib has a different profile and is particularly associated with pulmonary arterial hypertension. These differences show that vascular toxicity is not simply a class effect but depends on the additional pathways inhibited by each drug [39,40].

Several mechanisms may explain these effects. BCR-ABL inhibitors can alter endothelial survival, angiogenesis, permeability and vascular signaling. Ponatinib and dasatinib can also affect Rho-associated coiled-coil containing kinase (ROCK) signaling, which regulates endothelial structure and permeability. Increased ROCK activity has been associated with cardiovascular events in patients receiving second- and third-generation BCR-ABL inhibitors, while inhibition of this pathway can reverse some endothelial changes in experimental models. This provides a direct link between kinase inhibition, endothelial dysfunction and clinical vascular toxicity [41,42].

Bruton tyrosine kinase inhibitors (BTKIs) add another layer to this problem. Ibrutinib is associated with hypertension, atrial fibrillation, ventricular arrhythmias and bleeding. Newer agents such as acalabrutinib and zanubrutinib were developed to improve selectivity and generally have a more favorable cardiovascular profile, although cardiovascular events still occur. The role of the endothelium is less established than with BCR-ABL inhibitors, but BTK and related kinases are involved in pathways outside malignant B cells, including signaling relevant to vascular and platelet function. Endothelial effects may therefore contribute to the cardiovascular toxicity of these drugs together with direct effects on cardiac and platelet signaling [43,44].

Proteasome inhibitors are especially relevant in multiple myeloma. Carfilzomib is associated with hypertension, heart failure, ischemic events and other cardiovascular complications [45]. Endothelial dysfunction may contribute to this relatively broad toxicity profile. Proteasome inhibition can disturb endothelial nitric oxide signaling and vascular tone and cardiovascular toxicity can occur early after treatment begins [45,46]. Bortezomib generally has a lower cardiovascular risk than carfilzomib, supporting the idea that toxicity differs substantially even within the same treatment class [45].

Immunomodulatory drugs such as thalidomide, lenalidomide and pomalidomide have a different vascular profile. Their main cardiovascular concern is thrombosis, particularly when they are combined with corticosteroids or other anticancer treatments. In these patients, treatment-related effects are added to the already prothrombotic environment of multiple myeloma. The resulting thrombotic risk therefore cannot be explained by endothelial injury alone, but endothelial activation forms part of a wider interaction between the malignancy, coagulation system, platelets and treatment [47,48].

Immune-based treatments can produce a much more rapid form of endothelial activation. During CAR-T-cell therapy, activation of CAR-T cells and other immune cells leads to release of inflammatory cytokines and can trigger cytokine release syndrome (CRS). Endothelial cells respond to this inflammatory environment by becoming activated, more permeable and more procoagulant. Endothelial activation can then further amplify inflammation and vascular leakage. This creates a feedback loop between immune activation and endothelial injury rather than a simple one-way toxic effect [5,49].

This process is particularly important in severe CRS and immune effector cell-associated neurotoxicity syndrome (ICANS). Changes in angiopoietin signaling, von Willebrand factor, adhesion molecules, coagulation and vascular permeability have all been linked to severe toxicity after CAR-T therapy. Loss of endothelial barrier function may contribute to hypotension, capillary leak, tissue edema, coagulation abnormalities and organ dysfunction. The endothelium therefore acts not only as a target of the inflammatory response but also as an active part of CAR-T-related systemic toxicity [5,50].

Bispecific antibodies should also be considered in this context. These agents are now widely used in multiple myeloma, B-cell lymphomas and acute lymphoblastic leukaemia and, like CAR-T cells, can cause CRS through rapid immune-cell activation. Cardiovascular adverse events include hypotension, arrhythmias, heart failure, shock and coagulation abnormalities. Recent pharmacovigilance data have also identified signals for disseminated intravascular coagulation, myocarditis and fatal cardiovascular events, although the pattern differs between individual agents. Some cardiovascular events occur without documented CRS, which suggests that their cardiovascular toxicity may not be explained by cytokine release alone [51,52].

The endothelial mechanisms of bispecific antibody toxicity are not yet as well defined as those of CAR-T therapy. Cytokine-driven endothelial activation, increased vascular permeability, coagulation abnormalities and changes in vascular tone are plausible contributors, particularly during CRS. However, direct evidence remains limited, and it would be premature to describe endothelial injury as the main mechanism of cardiovascular toxicity from these drugs. Their rapid expansion in haematology makes this an important area for further study [53,54,55].

HSCT provides perhaps the clearest example of treatment-related endothelial injury because several vascular insults occur within a relatively short period. Conditioning regimens can directly damage endothelial cells before transplantation. This may be followed by exposure to calcineurin inhibitors and other immunosuppressive drugs, infections, inflammatory cytokines, complement activation and alloreactive immune responses. Previous anthracycline exposure and other cancer treatments may further reduce cardiovascular reserve before transplantation even begins [56,57].

Endothelial damage after HSCT is involved in sinusoidal obstruction syndrome, transplant-associated thrombotic microangiopathy, capillary leak syndrome and graft-versus-host disease. These conditions affect different organs and have different clinical presentations, but all involve loss of normal endothelial function to some degree. Cardiovascular complications after HSCT also extend beyond these early syndromes and include arrhythmias, heart failure, myocardial infarction and later vascular disease. The transplant setting therefore shows particularly clearly how repeated endothelial insults can connect acute treatment toxicity with longer-term cardiovascular risk [58,59].

**Figure 2:**
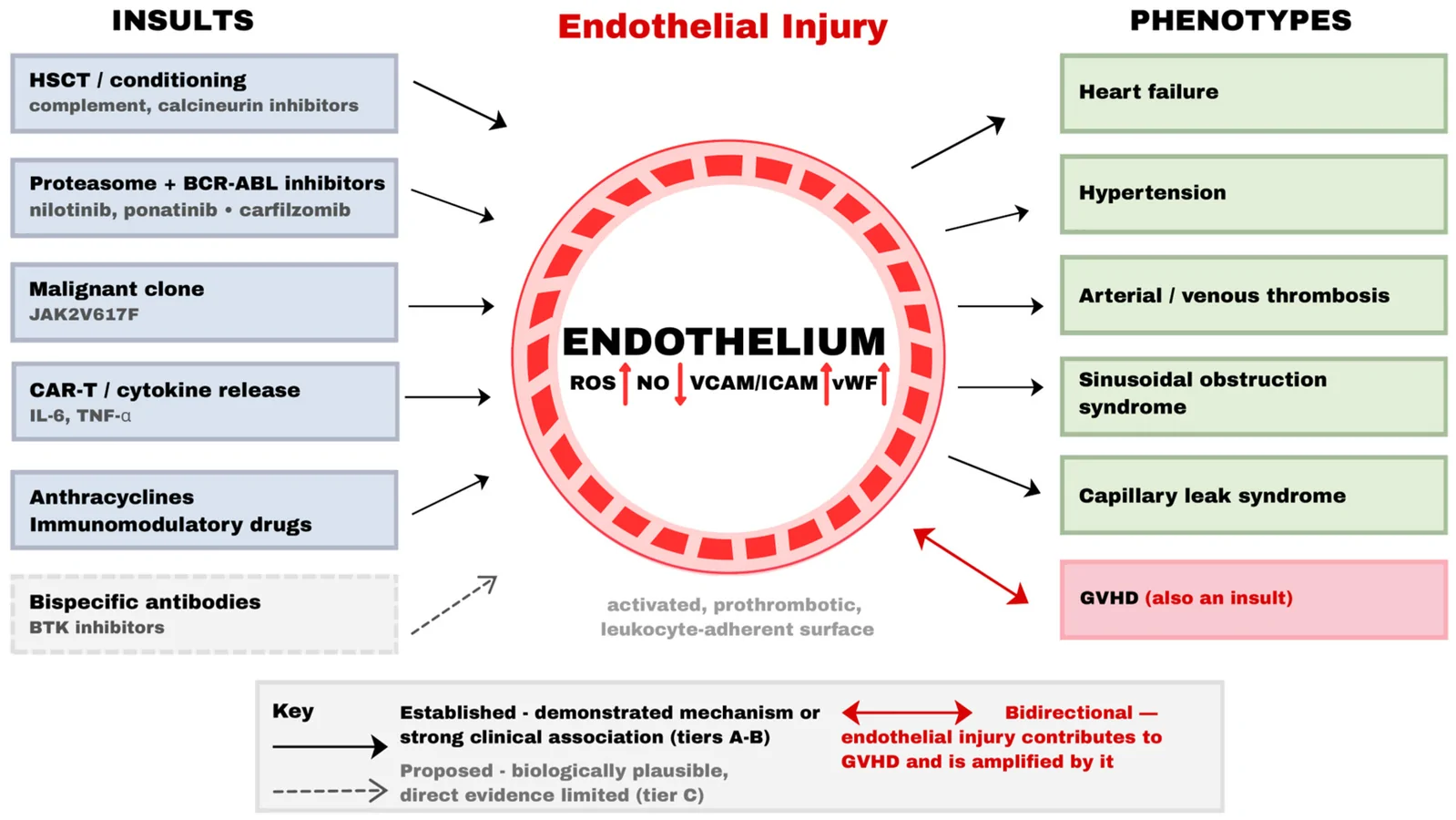
Endothelial injury as a potential contributor to cardiovascular toxicity in hematologic malignancies. Disease-related factors, anticancer therapies, immune activation and transplantation-related insults may converge on endothelial injury and contribute to different cardiovascular phenotypes. Solid arrows indicate demonstrated mechanisms or strong clinical associations, whereas dashed arrows indicate biologically plausible mechanisms with limited direct evidence. The bidirectional arrow illustrates the reciprocal relationship between endothelial injury and GVHD. HSCT, hematopoietic stem cell transplantation; CAR-T, chimeric antigen receptor T-cell; BTK, Bruton tyrosine kinase; ROS, reactive oxygen species; NO, nitric oxide; VCAM, vascular cell adhesion molecule; ICAM, intercellular adhesion molecule; vWF, von Willebrand factor; GVHD, graft-versus-host disease.

Overall, treatment-related endothelial injury should not be interpreted as a uniform mechanism across therapeutic classes. The most convincing evidence for a direct endothelial contribution is available for selected BCR-ABL inhibitors and HSCT. Evidence is moderate for anthracyclines and proteasome inhibitors, whereas the endothelial contribution to the cardiovascular effects of BTK inhibitors, immunomodulatory drugs and bispecific antibodies remains less clearly established. CAR-T-cell therapy is strongly associated with endothelial activation during severe immune-mediated toxicity, although its role in long-term cardiovascular complications remains uncertain. The treatment-specific pathways, their varying levels of supporting evidence and the bidirectional relationship between endothelial injury and GVHD are illustrated in Figure 2.

A comparative summary of the principal endothelial mechanisms, cardiovascular complications and strength of evidence across the discussed therapeutic classes is provided in Table 1.

## 5. Endothelial Injury as a Contributor to Cardiovascular Toxicity

Endothelial injury can lead to different cardiovascular complications depending on the vascular bed involved, the type of treatment and the patient’s underlying cardiovascular risk. This helps explain why endothelial dysfunction may present as hypertension in one patient, thrombosis in another and myocardial ischemia or dysfunction in others. These complications are therefore not always separate toxic effects but may share the same vascular origin. Cancer therapies associated with endothelial dysfunction have also been linked to arterial stiffening, impaired tissue perfusion, atherosclerotic disease and arterial thrombosis [60,,61].

One of the most direct consequences is hypertension. When the endothelium loses its ability to regulate vascular tone, vasoconstriction and peripheral vascular resistance increase; this effect can become more pronounced when treatment interferes with pathways involved in normal vascular signaling. The resulting rise in blood pressure is not only a clinical complication itself but can also increase cardiac afterload and make the myocardium more vulnerable to other treatment-related insults. In this way, vascular dysfunction and myocardial toxicity may develop together rather than as independent processes [61,62,63].

Thrombosis represents another important consequence of endothelial injury. A healthy endothelium normally limits platelet adhesion and coagulation, while an injured endothelium favors both. This shift can contribute to arterial and venous thrombotic events, especially when combined with the prothrombotic effects of the malignancy itself. The vascular toxicity of BCR-ABL tyrosine kinase inhibitors provides a clear example. Nilotinib and ponatinib are associated with arterial occlusive events, and experimental evidence shows that these agents can directly alter endothelial signaling and angiogenic function. Importantly, these effects differ between individual TKIs, suggesting that endothelial toxicity depends partly on the specific pathways inhibited by each drug [64,65].

Endothelial damage may also contribute to the development and progression of atherosclerotic disease. Persistent endothelial dysfunction favors vascular inflammation, platelet activation and abnormal interaction between circulating cells and the arterial wall. Over time, these changes may promote atherosclerosis or accelerate pre-existing vascular disease. This is particularly relevant in long-term survivors, in whom treatment-related vascular injury is added to traditional cardiovascular risk factors that accumulate with age. After HSCT, for example, survivors have an increased long-term burden of ischemic heart disease, stroke and other vascular diseases [66,67].

The coronary microcirculation provides another link between endothelial and myocardial injury. Impaired endothelial-dependent vasodilation can reduce coronary blood flow even when the epicardial coronary arteries are not obstructed [68]. Cancer therapy-related coronary microvascular dysfunction has been described after several treatment classes, including BCR-ABL TKIs and other antineoplastic therapies. It may contribute to myocardial ischemia, reduced coronary flow reserve and eventually myocardial dysfunction. Coronary microvascular dysfunction is also increasingly recognized as a possible mechanism behind clinical presentations such as ischemia with non-obstructive coronary arteries and heart failure [69,70].

Vascular injury can also increase arterial stiffness. A stiffer arterial system increases the pressure against which the left ventricle must pump and reduces the normal buffering function of large arteries. Arterial stiffening has been reported with several treatments relevant to hematologic malignancies, including BCR-ABL TKIs, proteasome inhibitors, chemotherapy and HSCT [71]. Together with hypertension and microvascular dysfunction, this can increase myocardial workload and may contribute to the development of cardiac dysfunction over time. These mechanisms are especially relevant after HSCT, where cardiovascular risk does not end with the early transplant period. Acute complications include heart failure, arrhythmias, thrombosis and pulmonary hypertension, while long-term survivors have an increased burden of heart failure and atherosclerotic disease. The endothelium may provide a link between the intense vascular injury that occurs around transplantation and some of the cardiovascular problems that appear years later, although this relationship is not yet fully understood [58,67].

Endothelial injury therefore provides a plausible link between hypertension, thrombosis, atherosclerotic disease, coronary microvascular dysfunction, arterial stiffness and myocardial dysfunction. The importance of this pathway probably differs between treatments and patients, and endothelial injury should not be considered the only mechanism of cardiovascular toxicity. However, viewing these complications through a common vascular pathway helps explain why different hematologic therapies can produce overlapping cardiovascular effects.

## 6. Endothelial Biomarkers and Cardiovascular Risk Stratification

Endothelial injury is difficult to measure directly, and no single biomarker can fully describe its severity. Several circulating markers can, however, capture different parts of the process. Angiopoietin-2 (Ang-2) reflects endothelial activation and vascular instability, while soluble thrombomodulin (sTM) may indicate loss of endothelial integrity. Increased levels of von Willebrand factor (vWF) and adhesion molecules like VCAM-1, ICAM-1 and E-selectin reflect endothelial activation and a shift toward a pro-inflammatory and prothrombotic state [72,73]. These markers have been studied particularly in patients undergoing HSCT, where higher levels are associated with endothelial complications and poorer outcomes [15,72,73]. A practical alternative is the Endothelial Activation and Stress Index (EASIX), calculated from lactate dehydrogenase, creatinine and platelet count. Although these variables are not specific to the endothelium, together they reflect cellular injury, renal involvement, platelet consumption and microangiopathic stress. EASIX has been associated with endothelial complications and adverse outcomes after allogeneic HSCT and has also been extended to CAR-T therapy [74,75,76]. Modified versions, particularly m-EASIX, replace creatinine with C-reactive protein to better capture the inflammatory component of CAR-T-related toxicity and have shown value in predicting severe CRS and ICANS [74].

Circulating biomarkers provide only part of the picture. Endothelial dysfunction can also be assessed through glycocalyx integrity, microvascular perfusion, flow-mediated dilation and arterial stiffness. Combining these functional measurements with circulating markers may be more informative than relying on either approach alone, especially because endothelial injury is heterogeneous and may differ between vascular beds. Recent evidence supports this multimodal approach, with relationships observed between EASIX, Ang-2, glycocalyx impairment and measures of tissue perfusion [77].

For cardiovascular risk assessment, however, an important gap remains. Most available endothelial markers have been evaluated as predictors of acute HSCT or CAR-T complications and treatment-related mortality, rather than later cardiovascular disease. Their ability to predict hypertension, thrombosis, coronary disease, microvascular dysfunction, or heart failure therefore remains uncertain [77,78].

A useful future approach may be to combine endothelial markers with established cardiac biomarkers such as troponin and natriuretic peptides. Rather than replacing conventional cardiac monitoring, endothelial assessment could identify the vascular component of toxicity and potentially detect risk before overt cardiovascular injury becomes clinically apparent.

The biological relevance, investigated populations, predictive outcomes, specificity and current level of clinical validation of the principal endothelial markers are summarized in Table 2.

## 7. Endothelial Protection as a Therapeutic Strategy

If endothelial injury contributes to cardiovascular toxicity, preservation of vascular function may represent a relevant component of cardio-oncology care. However, no intervention has currently been established specifically for the prevention of endothelial injury across different haematologic malignancies and therapies. Current clinical practice is therefore based primarily on the assessment and management of modifiable cardiovascular risk factors, the use of established cardioprotective treatments when clinically indicated and closer monitoring of patients at increased cardiovascular risk. Current recommendations emphasize the identification and management of hypertension, dyslipidaemia, diabetes, smoking and other cardiovascular risk factors before, during and after potentially cardiotoxic treatment [39,58].

Endothelial protection should be distinguished from general cardioprotection. General cardioprotection includes strategies intended to prevent or reduce cardiovascular injury broadly, particularly myocardial dysfunction, heart failure, hypertension and arrhythmias. In contrast, endothelial protection specifically aims to preserve endothelial integrity and function, including nitric oxide signalling, vascular barrier stability, vasodilation and the normal anti-inflammatory and antithrombotic properties of the vascular surface. The two concepts overlap because reducing blood pressure, inflammation or oxidative stress may benefit both the myocardium and the vasculature [79]. However, an intervention should not be described as endothelial-protective unless its effects on endothelial function or endothelial injury-related outcomes have been directly demonstrated.

Statins are of interest because, in addition to lowering lipid levels, they have anti-inflammatory and antioxidant effects that may help preserve endothelial function. In patients with lymphoma receiving anthracycline-based chemotherapy, atorvastatin reduced the risk of a clinically relevant decline in left ventricular function. Other data suggest that atorvastatin may reduce anthracycline-related aortic stiffening and preserve aortic distensibility. Although these findings are compatible with a vascular protective effect, they do not establish endothelial protection as the mechanism responsible for the observed clinical benefit. Also, results from statin trials across broader cancer populations have been inconsistent. Therefore, the available evidence does not support the routine use of statins solely for endothelial protection in all patients receiving anthracyclines [80,81].

ACE inhibitors, angiotensin receptor blockers and beta-blockers are widely used in cardio-oncology, particularly in patients with hypertension, established cardiovascular disease or an increased risk of myocardial dysfunction. Their clinical evidence relates mainly to blood pressure control and the prevention or treatment of myocardial dysfunction rather than direct endothelial protection. Adequate blood pressure control is especially important when anticancer treatment increases vascular resistance or causes hypertension. However, whether these drugs prevent treatment-related endothelial injury independently of their established cardiovascular effects remains uncertain [39,63].

Treatment-specific approaches can also reduce cardiovascular injury. In patients at increased risk who require anthracyclines, dexrazoxane and liposomal formulations of doxorubicin may reduce cardiac exposure and myocardial toxicity. These interventions are established or supported for the prevention of anthracycline-related cardiac injury, but they should not be considered specific endothelial-protective therapies. Any beneficial effect on endothelial function remains secondary or insufficiently established [39,82,83].

Defibrotide represents the most direct clinically established treatment targeting an endothelial injury-related complication. It has anti-inflammatory, antithrombotic, fibrinolytic and endothelial-protective properties and is used for severe hepatic veno-occlusive disease/sinusoidal obstruction syndrome following HSCT. Its potential use in other endothelial injury-related conditions, including transplant-associated thrombotic microangiopathy, graft-versus-host disease and complications of CAR-T-cell or bispecific antibody therapy, remains investigational. Current evidence does not support its routine use for these indications or for general cardiovascular protection [16,84].

Lifestyle measures are recommended as part of general cardiovascular risk reduction. Regular physical activity, smoking cessation and management of obesity, diabetes and dyslipidaemia can improve blood pressure, metabolic health and cardiorespiratory fitness and may support endothelial function. These measures are particularly relevant in long-term survivors, in whom conventional cardiovascular risk factors may accumulate alongside treatment-related vascular injury. Nevertheless, direct evidence that lifestyle interventions specifically prevent endothelial injury caused by haematologic therapies remains limited [39,85,86].

At present, endothelial biomarkers, EASIX, arterial stiffness and measures of microvascular function should be regarded primarily as investigational tools rather than as established guides to treatment. They may eventually help identify vascular injury before clinically apparent cardiovascular disease and support more individualized preventive strategies. However, prospective studies are needed to determine whether biomarker- or vascular function-guided interventions improve clinical outcomes before these approaches can be incorporated into routine cardio-oncology care.

## 8. Current Knowledge Gaps and Future Research Directions

Many questions about endothelial injury in hematologic malignancies remain unanswered. One of the most important is when the injury actually begins. Some patients may already have endothelial dysfunction because of the malignancy itself, while treatment adds further damage. Studies that measure endothelial function before treatment, during therapy and after treatment could help separate these effects. They could also show whether endothelial function returns to normal or whether some changes remain for months or years.

It is also unclear why some patients develop cardiovascular complications while others receiving the same treatment do not. Age, hypertension, diabetes, dyslipidaemia, previous cardiovascular disease and earlier cancer treatments may all affect how the endothelium responds. The malignancy itself may also be important. These factors should be studied together to identify patients who are more likely to develop vascular injury.

Another question is whether early endothelial injury leads to cardiovascular disease later. Most studies have focused on complications that occur during treatment or shortly afterwards. Longer follow-up is needed to determine whether vascular changes during treatment are followed by hypertension, thrombosis, coronary disease, microvascular dysfunction or heart failure. This is especially important after HSCT, where cardiovascular disease may appear years after transplantation.

There is also little information about the long-term vascular effects of newer treatments. CAR-T cells and bispecific antibodies can cause marked endothelial activation during acute toxicity, but it is not known whether these changes completely resolve after recovery. The same problem exists with treatments that may be given for long periods, such as BCR-ABL and BTK inhibitors. We need to know whether repeated or prolonged exposure can produce lasting vascular damage.

Finally, it remains unclear whether detecting endothelial injury early can actually improve patient care. Future studies should test whether patients with early signs of vascular injury benefit from closer cardiovascular monitoring, changes in cancer treatment or earlier preventive therapy. If these approaches reduce later cardiovascular complications, endothelial assessment could become useful in routine cardio-oncology care.

### Limitations

This review has several limitations. It is narrative rather than systematic and does not include a predefined search strategy, formal study selection process or assessment of study quality. Therefore, relevant evidence may have been omitted, and the selection and interpretation of sources may be subject to bias. The evidence across the discussed domains is heterogeneous, ranging from demonstrated mechanisms and strong clinical associations to biologically plausible or speculative hypotheses based mainly on observational, indirect or experimental findings. The proposed framework should therefore be regarded as a clinically oriented synthesis intended to guide discussion and future research rather than as a definitive evidence-based guideline.

## 9. Conclusions

Endothelial injury represents an important component of cardiovascular toxicity in haematologic malignancies, but it is not the only mechanism involved. Vascular injury may begin with the malignancy itself and be further amplified by anticancer therapy, immune activation, transplantation and pre-existing cardiovascular risk factors. These processes can contribute to hypertension, thrombosis, ischaemia, microvascular dysfunction, arterial stiffness and myocardial dysfunction.

The relative importance of endothelial injury varies across diseases and treatments. Its contribution is supported most clearly in settings such as BCR-ABL inhibitor therapy, CAR-T-cell-related toxicity and HSCT, whereas evidence for a direct endothelial role remains more limited for other therapies. Direct cardiomyocyte injury, electrophysiological effects, platelet dysfunction, systemic inflammation and conventional cardiovascular risk factors may act independently or together with endothelial dysfunction.

Endothelial biomarkers and vascular assessments may eventually improve risk stratification and support earlier preventive interventions. However, their specificity, clinical validation and ability to predict cardiovascular outcomes remain limited. Further prospective studies are required before endothelial assessment or endothelial-directed treatment can be incorporated into routine cardio-oncology care.

## Figures and Tables

**Table 1:** Endothelial mechanisms and cardiovascular complications associated with therapies used in hematologic malignancies.

Therapeutic Class or Approach	Principal Endothelial Mechanisms	Main Cardiovascular Complications	Evidence for Endothelial Involvement
Anthracyclines	Direct endothelial injury, oxidative stress, reduced NO bioavailability, endothelial apoptosis and senescence, and impaired vasodilation	Myocardial dysfunction and heart failure, with possible coronary microvascular dysfunction and arterial stiffening	Moderate; supported by experimental and clinical vascular evidence, although direct cardiomyocyte injury remains the dominant established mechanism
BCR-ABL tyrosine kinase inhibitors	Impaired endothelial survival and angiogenesis, altered permeability and vascular signalling, and dysregulation of ROCK signalling	Arterial occlusive events, myocardial infarction, stroke and peripheral arterial disease, particularly with nilotinib and ponatinib; pulmonary arterial hypertension with dasatinib	Strong; supported by mechanistic, experimental and clinical evidence, with important differences among individual agents
Bruton tyrosine kinase inhibitors	Possible effects on vascular and platelet signalling; direct endothelial involvement remains incompletely defined	Hypertension, atrial fibrillation, ventricular arrhythmias and bleeding, particularly with ibrutinib	Limited; cardiovascular associations are well established, but the specific contribution of endothelial injury remains uncertain
Proteasome inhibitors	Disruption of endothelial NO signalling and vascular tone, with consequent endothelial dysfunction	Hypertension, heart failure and ischaemic events, particularly with carfilzomib	Moderate; clinical toxicity is well documented, and endothelial dysfunction is biologically supported, but other cardiac mechanisms also contribute
Immunomodulatory drugs	Endothelial activation within a broader interaction involving malignancy-related hypercoagulability, platelets and coagulation pathways	Venous and arterial thrombosis, particularly when combined with corticosteroids or other anticancer therapies	Moderate for thrombotic risk but limited for endothelial injury as an independent mechanism
CAR-T-cell therapy	Cytokine-driven endothelial activation, increased vascular permeability, procoagulant transformation and impaired endothelial barrier function	Hypotension, capillary leak, coagulation abnormalities, arrhythmias, myocardial dysfunction and organ injury during severe CRS and ICANS	Strong for acute endothelial activation during severe CRS and ICANS; evidence for long-term vascular effects remains limited
Bispecific antibodies	Cytokine-mediated endothelial activation, increased permeability, altered vascular tone and coagulation abnormalities, particularly during CRS	Hypotension, arrhythmias, heart failure, shock and coagulation abnormalities	Limited; mechanisms are plausible and supported indirectly, but direct endothelial evidence remains insufficient
Haematopoietic stem cell transplantation	Conditioning-related endothelial damage combined with inflammation, complement activation, immune injury, infections, immunosuppressive therapy and coagulation abnormalities	Sinusoidal obstruction syndrome, transplant-associated thrombotic microangiopathy, capillary leak syndrome, hypertension, thrombosis, arrhythmias, heart failure and long-term vascular disease	Strong; supported by extensive mechanistic and clinical evidence, particularly for acute endothelial complications

Note: The strength of evidence represents a qualitative synthesis based on the directness and consistency of the experimental and clinical evidence discussed in this review and does not constitute a formal evidence-grading assessment. NO, nitric oxide; ROCK, Rho-associated coiled-coil containing kinase; CRS, cytokine release syndrome; ICANS, immune effector cell-associated neurotoxicity syndrome.

**Table 2:** Potential biomarkers and assessment tools for endothelial injury in haematologic malignancies.

Marker or Assessment	What It Reflects	Evidence Population	Reported or Potential Outcome Predicted	Specificity	Clinical Validation
Angiopoietin-2 (Ang-2)	Endothelial activation, vascular instability and increased permeability	Predominantly patients undergoing HSCT; also studied in cellular therapy	Endothelial complications, severe treatment-related toxicity and adverse outcomes	Limited; may also increase with systemic inflammation and critical illness	Investigational; not validated for routine cardiovascular risk prediction
Soluble thrombomodulin (sTM)	Loss of endothelial integrity and endothelial-cell injury	Predominantly HSCT populations	Endothelial complications and adverse transplant outcomes	Limited; influenced by systemic inflammation, coagulation abnormalities and organ dysfunction	Investigational; no established clinical threshold for cardiovascular monitoring
von Willebrand factor (vWF)	Endothelial activation and a prothrombotic endothelial phenotype	HSCT, CAR-T therapy and other haematologic settings	Severe toxicity, coagulation abnormalities and endothelial complications	Low; affected by inflammation, thrombosis, age and other cardiovascular conditions	Research use; not independently validated for routine risk stratification
VCAM-1, ICAM-1 and E-selectin	Endothelial inflammatory activation and leukocyte adhesion	Mainly HSCT and experimental or translational studies	Endothelial complications and severity of inflammatory toxicity	Low; elevated in many inflammatory and vascular disorders	Investigational; clinical thresholds and standardized application are lacking
EASIX	Combined microangiopathic stress, cellular injury, renal dysfunction and platelet consumption	Primarily allogeneic HSCT	Endothelial complications, treatment-related mortality and adverse transplant outcomes	Low; components are not endothelial-specific and are influenced by renal, haematologic and systemic factors	Externally studied in HSCT, but not validated as a specific cardiovascular prediction tool
Modified EASIX (m-EASIX)	Inflammatory and microangiopathic stress	CAR-T-cell therapy	Severe CRS and ICANS	Low; strongly influenced by systemic inflammation and cytopenia	Promising for acute toxicity prediction, but not established for routine cardiovascular monitoring
Glycocalyx assessment	Structural damage to the endothelial surface layer	Predominantly HSCT and cellular-therapy populations	Endothelial dysfunction and treatment-related complications	Moderate biological relevance but limited disease specificity	Experimental; methods and thresholds are not standardized
Flow-mediated dilation	Endothelium-dependent vascular function	Limited oncology and treatment-exposed populations	Subclinical vascular dysfunction and potential later cardiovascular risk	Limited; affected by conventional cardiovascular risk factors and measurement conditions	Established vascular research method, but not validated for routine use in haematologic malignancies
Arterial stiffness	Functional and structural vascular impairment	Cancer survivors and patients exposed to chemotherapy, TKIs or HSCT	Vascular ageing and possible long-term cardiovascular risk	Low; affected by age, blood pressure and pre-existing cardiovascular disease	Clinically measurable, but its predictive role in this setting remains insufficiently validated

## Data Availability

No new data were created or analyzed in this study. Data sharing is not applicable to this article.
